# Integrative mRNA and Long Noncoding RNA Analysis Reveals the Regulatory Network of Floral Bud Induction in Longan (*Dimocarpus longan* Lour.)

**DOI:** 10.3389/fpls.2022.923183

**Published:** 2022-06-14

**Authors:** Fan Liang, Yiyong Zhang, Xiaodan Wang, Shuo Yang, Ting Fang, Shaoquan Zheng, Lihui Zeng

**Affiliations:** ^1^Insititute of Genetics and Breeding in Horticultural Plants, College of Horticulture, Fujian Agriculture and Forestry University, Fuzhou, China; ^2^Fujian Breeding Engineering Technology Research Center for Longan & Loquat, Fruit Research Institute, Fujian Academy of Agricultural Sciences, Fuzho, China

**Keywords:** longan, transcriptome, mRNA, long noncoding RNA, floral bud induction, WGCNA

## Abstract

Longan (*Dimocarpus longan* Lour.) is a tropical/subtropical fruit tree of significant economic importance. Floral induction is an essential process for longan flowering and plays decisive effects on the longan yield. Due to the instability of flowering, it is necessary to understand the molecular mechanisms of floral induction in longan. In this study, mRNA and long noncoding RNA (lncRNA) transcriptome sequencing were performed using the apical buds of fruiting branches as materials. A total of 7,221 differential expressions of mRNAs (DEmRNAs) and 3,238 differential expressions of lncRNAs (DElncRNAs) were identified, respectively. KEGG enrichment analysis of DEmRNAs highlighted the importance of starch and sucrose metabolic, circadian rhythms, and plant hormone signal transduction pathways during floral induction. Combining the analysis of weighted gene co-expression network (WGCNA) and expression pattern of DEmRNAs in the three pathways, specific transcriptional characteristics at each stage during floral induction and regulatory network involving co-expressed genes were investigated. The results showed that sucrose metabolism and auxin signal transduction may be crucial for the growth and maturity of autumn shoots in September and October (B1-B2 stage); starch and sucrose metabolic, circadian rhythms, and plant hormone signal transduction pathways participated in the regulation of floral bud physiological differentiation together in November and December (B3-B4 stage) and the crosstalk among three pathways was also found. Hub genes in the co-expression network and key DEmRNAs in three pathways were identified. The circadian rhythm genes *FKF1* and *GI* were found to activate *SOC1*gene through the photoperiod core factor *COL* genes, and they were co-expressed with auxin, gibberellin, abscisic acid, ethylene signaling genes, and sucrose biosynthesis genes at B4 stage. A total of 12 hub-DElncRNAs had potential for positively affecting their distant target genes in three putative key pathways, predominantly in a *co*-transcriptional manner. A hypothetical model of regulatory pathways and key genes and lncRNAs during floral bud induction in longan was proposed finally. Our studies will provide valuable clues and information to help elucidate the potential molecular mechanisms of floral initiation in longan and woody fruit trees.

## Introduction

Longan (*Dimocarpus longan* Lour.), which belongs to the family of Sapindaceae, is a very important horticultural crop in the tropical and subtropical regions of the world. Floral bud induction largely determines the fruit yield of the next year (Link, 2000). However, the difficulty and instability of blossoming are the most challenging issues in longan production because the apical buds are easily disturbed by various factors during floral induction (You et al., 2012). Therefore, a lot of attention has been paid to the regulatory mechanism of floral induction in longan.

Floral induction is critical in the life cycle of plants, which is influenced by a variety of endogenous and environmental stimuli (Wang and Ke, 1992; Kurokura et al., 2013; Lyons et al., 2015). In the model plant *Arabidopsis*, the molecular mechanisms of flowering have been well established, including vernalization, photoperiod, phytohormones, nutrient responses, and senescence pathways (Wang et al., 2009; Kim et al., 2012; Yamaguchi and Abe, 2012; Cho et al., 2017). Among them, photoperiod and temperature are primary environmental cues. In the photoperiod regulatory pathway, the activity of CONSTANS (CO) protein is tightly controlled by the circadian clock and light signals, thereby affecting integrated genes, such as *FLOWERING LOCUS T* (*FT*) and *SUPPRESSOR OF OVEREXPRESSION OF CONSTANS1* (*SOC1*), to accelerate the flowering process (Pin and Nilsson, 2012). Unlike annual plants, which bloom only once in their life cycle, perennial woody plants can live for many years and flower repeatedly (Albani et al., 2012). However, little is known about the regulatory mechanism of floral bud induction seasonally in woody plants, especially in longan.

Sugars are important endogenous signals in the regulation of plant growth and development, including flowering (Turnbull, 2011). It has been reported that the flowering transition of citrus was regulated by sugar as an energy substance (Shalom et al., 2014). Previous research studies have revealed that T6P, glucose, and sucrose mediated the regulation of floral induction as signaling molecules (Wahl et al., 2013). Sucrose, glucose, and starch are closely related to photoperiod and abscisic acid (ABA) signal and play an indispensable role in floral induction (Su et al., 1997; Curaba et al., 2014; Han et al., 2014). *AtIDD8*, a transcription factor associated with sugar signaling, has been demonstrated to regulate photoperiodic flowering by regulating sugar transport and metabolism in *Arabidopsis* (Seo et al., 2011). The expression of the *CO* gene is affected by the expression of *GBSSI*, a key gene of starch synthase, to promote plant flowering (Serrano et al., 2009).

Phytohormones are also important factors for floral induction. Plant endogenous hormones can regulate plant growth and development by binding to specific protein receptors, and play a micro-efficient regulatory role in the floral bud differentiation of woody plants (Su et al., 1997; Mutasa-Gottgens and Hedden, 2009; Curaba et al., 2014; Han et al., 2014). Gibberellin (GAs) has a positive effect on controlling the onset of flower formation in *Arabidopsis* (Yamaguchi et al., 2014). Physiological and biochemical evidence indicates that auxin and GAs play essential roles in apple floral induction (Xing et al., 2015). Cytokinin (CK) levels have been reported to affect meristem activity and inflorescence complexity in *Arabidopsis* and *Oryza sativa* (Han et al., 2014). Stress hormones, such as abscisic acid (ABA), jasmonic acid (JA), and ethylene (ETH), participate in flowering regulation under abiotic stress through the flowering pathway related to photoperiod and circadian clock (de Montaigu et al., 2010; Shimakawa et al., 2012; Lyons et al., 2015). In fruit trees, hormone's effects on flowering are quite different from model plants, indicating that there may be a more complex regulatory mechanism in fruit trees.

Some flowering genes have been identified in longan. Photoperiodic flowering pathway–related genes *DlGI* and *DlFKF1* were found to induce the flowering of longan by affecting the synthesis and transportation of endogenous auxin (Huang et al., 2017). Both *DlELF4-1* and *DlELF4-2* transgenic *Arabidopsis* plants showed delayed flowering and adventitious root growth, suggesting that they may inhibit flowering and promote auxin synthesis (Fu et al., 2018). These studies suggest that phytohormone and photoperiod may be interrelated in the regulation of flowering in longan.

Another potential regulatory factor in floral induction is long noncoding RNAs (lncRNAs), which are a class of RNA transcripts over 200 nt in length that lack significant protein-coding capacity (Jin et al., 2013). LncRNAs usually have a similar structure to mRNAs. Advancements in lncRNA research have shown that most transcribed regions generate lncRNAs, which may have rich features, not as “transcriptional noise” (i.e., no biological functions). In plants, lncRNAs are involved in diverse biological processes, including flowering and phytohormone-related development (Liu et al., 2015). LncRNAs have been reported in a variety of plant species, including *Arabidopsis thaliana* (Yuan et al., 2016), *Cucumis sativus* (Hao et al., 2015), *Oryza sativa* (Huang et al., 2021), and *Citrus sinensis* (Ke et al., 2019). In *Arabidopsis*, two different classes of lncRNAs (*COOLAIR* and *COLDAIR*) are involved in flowering regulation by repressing *FLOWERING LOCUS C* (*FLC*) *via* epigenetic modulation, respectively (Heo and Sung, 2011; Sun et al., 2013). In rice, *Early flowering-completely dominant* (*Ef-cd*) encoding an antisense lncRNAOsSOC1 overlapping a floral activator shortens maturity without concomitant yield loss (Fang et al., 2019). It is suggested that flowering genes and related lncRNAs may play a joint role in floral induction and flowering in plants. However, little is known about lncRNAs and their potential roles during floral induction in fruit trees.

The purpose of this study is to explore the transcriptional regulation of mRNA and lncRNA during floral induction in longan. DEmRNAs and DElncRNAs related to floral bud initiation were identified, and the *cis-* and *trans*-target genes of DElncRNAs were systematically predicted and analyzed. The potential relationship between mRNA and lncRNA during longan floral induction was analyzed. Furthermore, regulatory networks and key candidate genes in response to longan floral bud induction were identified by constructing co-expression networks. Our results will reveal novel insights into the regulatory mechanisms underlying seasonal flowering induction in longan and provide strategies for increasing longan yield.

## Materials and Methods

### Plant Materials

In the current study, apical buds of fruiting branches (diameter over 0.8 cm) of “Lidongben” (subsequently referred to as “LDB”) longan from September 2019 to January 2020 were used as experimental materials. The mature “LDB” trees grow in the experimental field of Fruit Tree Research Institute of Fujian Academy of Agricultural Sciences. The apical buds of five stages (B1, September; B2, October; B3, November; B4, December; B5, January) were collected. Samples were collected from 10 am to 12 am on the 20th of each month and each stage had three biological replicates. After collection, the buds were immediately frozen in liquid nitrogen and stored at −80°C until used. According to China Weather Network (http://www.weather.com.cn/jiangsu), the climate data of Fuzhou from September 2019 to January 2020 was recorded in Supplementary Table 1.

### Library Preparation for Transcriptome Sequencing and Identification of mRNA and lncRNA

A total amount of 1 μg RNA per sample was used as input material for the RNA sample preparations. Sequencing libraries were generated using NEBNext UltraTM RNA Library Prep Kit for Illumina (NEB, United States) following the manufacturer's recommendations. The library preparations were sequenced on an Illumina platform and paired-end reads were generated. All clean reads generated in this study were deposited in the NCBI Sequence Read Archive database (http://www.ncbi.nlm.nih.gov/sra/) under the project accession number PRJNA830603.

Annotated mRNAs were obtained by comparison with CPC2^1^ / PFAM^2^ / CNCI^3^ databases using Cuffcompare software. Cuffmerge software was used to merge the transcripts obtained by the splicing of all samples, and removed the transcripts with uncertain chain directions and transcripts with a length of less than 200 nt. Next, Cuffcompare software was used to screen out transcripts that overlapped with the exon region annotated in the database, and included the lncRNAs in the database that overlapped with the exon region of the spliced transcripts as database annotation lncRNAs in the subsequent analysis. The transcripts without coding potential were lncRNAs. For the transcripts obtained in the previous step, the current mainstream coding potential analysis databases (CPC2 / PFAM / CNCI) were combined to predict the coding potential, and the intersection of the transcripts with coding potential in these software results was taken as the Novel_mRNA predicted by this analysis. Take the intersection of transcripts without coding potential in these software results as the candidate Novel_lncRNA data set predicted by this analysis (Harrow et al., 2012). According to the naming guidelines of HGNC^4^ (Povey et al., 2001), the candidate novel_lncRNA was finally screened and named according to its positional relationship with coding genes. The lncRNA sequences of *Malus domestica, Citrus sinensis*, and *Arabidopsis thaliana* were obtained from the Green Non-coding Database^5^ (GREENC) (Paytuvi Gallart et al., 2016) for sequence conservation analysis. Conservation analysis of lncRNAs was performed using the BLAST tool of integrative toolkit TBtools (Chen et al., 2020).

### Analysis of DEmRNAs and DElncRNAs

Fragments per kilobase million (FPKM) were used to quantify mRNAs and lncRNAs expression levels in each sample using StringTie software. Screening and identification of DEmRNAs and DElncRNAs using edgeR software (Robinson et al., 2010). The DEmRNAs and DElncRNAs were identified based on the following parameters: |log_2_foldchange| ≥ 1 and *p* < 0.05. Kyoto Encyclopedia of Genes and Genomes (KEGG) enrichment analyses were performed using DEmRNAs that were differentially combined in different stages.

### Target Gene Prediction of lncRNA

There are various mechanisms by which lncRNAs regulate target genes, and the most common ways to target genes are *cis* and *trans* target gene regulation. According to the positional relationship between lncRNA and mRNA, the protein-coding gene transcribed within 100 kb upstream and downstream of the lncRNA was identified as the *cis* target gene (Bao et al., 2019). In addition, *trans* target genes were determined by calculating the correlation between lncRNAs and mRNAs expression levels, and the correlation of expression was assessed using Pearson's correlation coefficient (*r* > 0.95) (Kopp and Mendell, 2018). The log_2_transformed (FPKM + 1) value was used to normalize the expression levels of mRNA and lncRNA. The expression levels of DEmRNAs and DElncRNAs were heatmapped with integrative toolkit TBtools on the basis of normalized FPKM values. LncRNA sequences were used as queries to discover the enriched motifs using online server STREME^6^ (Bailey, 2021) (*p* < 0.05). The possible roles of each identified motif was predicted using online server GOMO (gene ontology for motifs^7^) (Buske and Bodén, 2010). In the MEyellow and MEblue modules, transcription factors (TFs) were identified and classified according to the reference Plant TFDB 4.0 database^8^.

### Coexpression Network Analysis of DEmRNAs and DElncRNAs

R package WGCNA (Zhang and Horvath, 2005; Langfelder and Horvath, 2008) was used to test whether the expression levels of DEmRNAs and DElncRNAs were correlated with floral bud induction and the association between DEmRNAs and DElncRNAs. In this analysis, the soft threshold power was set to 5, and then an adjacency matrix was constructed using the adjacency function. Based on the transformation of the adjacency matrix into a topological overlap metric graph, similarity matrices of DEmRNAs and DElncRNAs expression between different nodes were calculated. Based on this algorithm, DEmRNAs and DElncRNAs were hierarchically clustered and modules were defined. As the method described by Gerttula et al. (2015), the modules were associated with stages and the correlation matrix of both was computed. The module with Pearson's correlation coefficient >0.75 or < −0.5 and *p* < 0.05 were designated as the module most correlated with the stages. The module membership (MM) value of the co-expression network for each module was calculated and used to rank members in each module and identify hub-genes according to MM > 0.75. On this basis, the network of co-expression centers was analyzed and mapped by Cytoscape (version 3.7.1) (Jiang et al., 2017).

### Expression Validation of Candidate Gene by qRT-PCR

Total RNA was isolated and extracted from longan apical buds using RNAprep Pure Plant Plus Kit (TIANGEN, China), and was reverse transcribed into cDNA with *5*×*TransScript* Uni All-in-One SuperMix and gDNA Remover in the *PerfectStart* Uni RT&qPCR Kit (Transgen, China) according to the manufacturers' instructions. qRT-PCR was performed on the instrument of *LightCycler 96* PCR System (Roche, Switzerland) using *PerfectStart* Green qPCR SuperMix (Transgen,China). The 2^−ΔΔCt^ method was used to calculate the relative expression levels of candidate genes (Jue et al., 2018). The actin gene *DlACT* (Supplementary Table 2) was used as the internal reference to normalize expression levels. The primer sequence information was shown in Supplementary Table 2. Three biological replicates were used for each experiment.

## Results

### Development of Apical bud During Floral Induction in Longan

**Figure 1:**
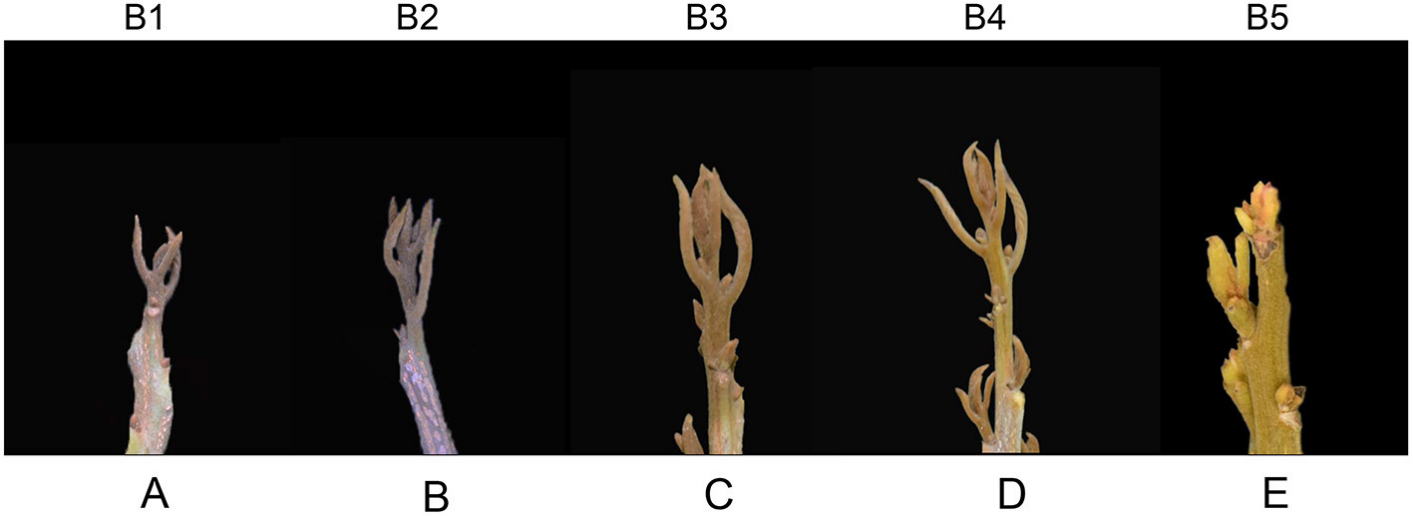
Morphological characteristics of the apical bud during floral bud induction in longan. **(A–E)** Morphological characteristics of apical buds at B1, B2, B3, B4, and B5 stages, respectively.

In Fujian Province, autumn shoots of longan grow and get maturity in September (B1 stage) and October (B2 stage). Then in November and December (B3, B4 stage), apical buds of fruiting branches turn into the stage of floral bud physiological differentiation, followed by morphological differentiation in January (B5 stage) (Wang and Ke, 1992; Yuan, 2016). The phenotypes of apical buds of “LDB” longan at B1, B2, B3, B4, and B5 stages were shown in Figure 1. The apical buds at B1 (Figure 1A) and B2 (Figure 1B) stages were shorter overall compared to other stages. The axis of the apical bud is elongated and axillary buds can be seen in the axil at B3 and B4 stages (Figures 1C,D). At the B5 stage, the apical buds turned red (called “red bud”) (Figure 1E).

### Characterization of mRNA and lncRNA

**Figure 2:**
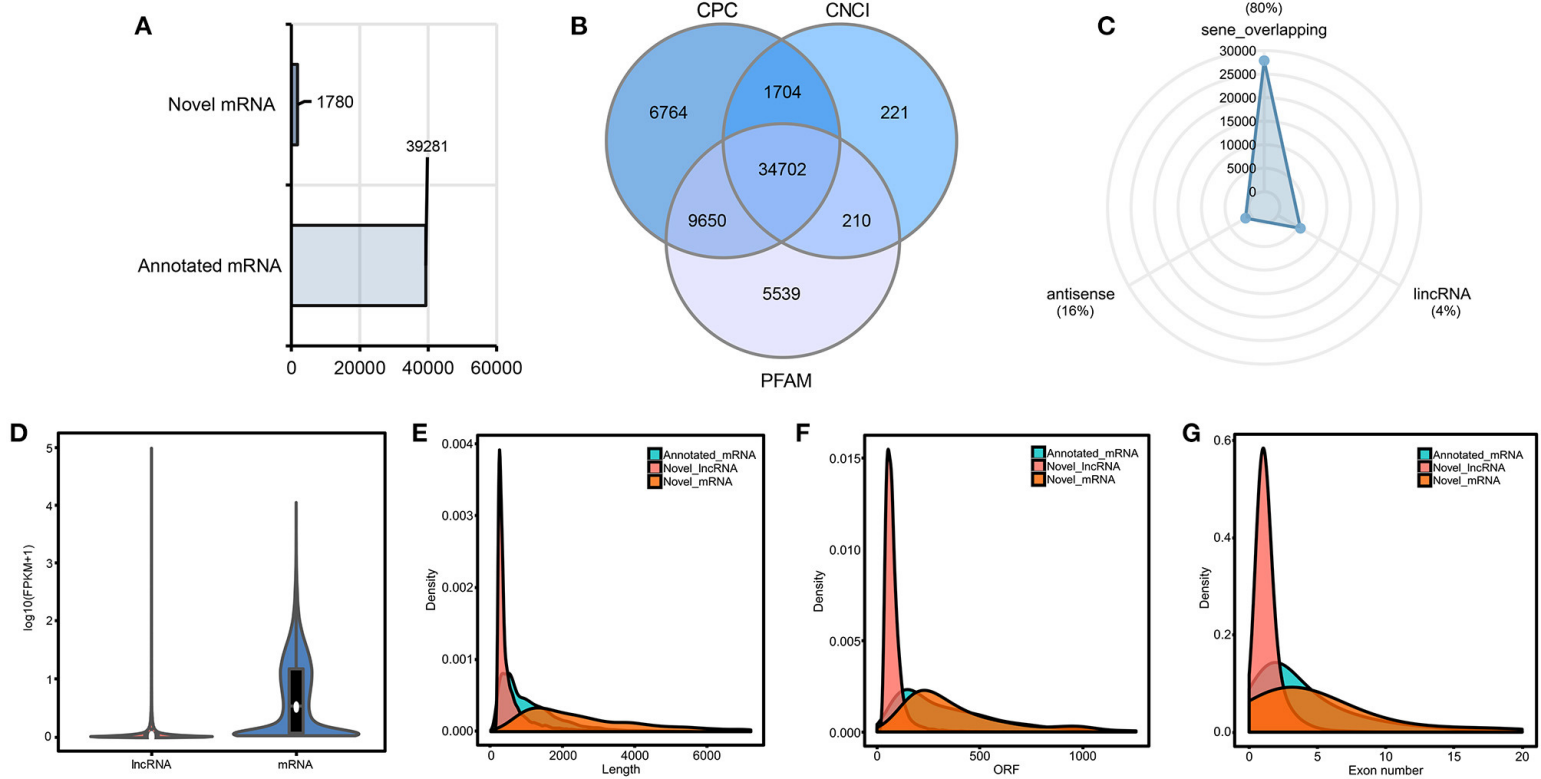
Characterization and statistical analysis of candidate mRNAs and lncRNAs. **(A)** Statistics of novel mRNA and annotated mRNA. The horizontal axis indicates the number of mRNAs. **(B)** Venn diagram of candidate lncRNAs. **(C)** Classification of the identified lncRNAs. **(D)** FPKM distribution of mRNA and lncRNA transcripts. **(E–G)** Characterization of annotated mRNA, novel mRNA, and novel lncRNA transcripts. The horizontal axis of the density plot from left to right indicates total length (left), ORF length (middle), and the number of exons (right) of annotated mRNA, novel mRNA, and novel lncRNA transcripts, respectively.

Through genomic comparison, CPC, CNCI, and PFAM database analysis, 39,281 annotated mRNAs, 1,780 novel mRNAs (Figure 2A), and 34,702 putative lncRNAs (Figure 2B) were identified (Supplementary Table 3). The most abundant class was seen overlapping lncRNAs (80%), followed by antisense lncRNAs (16%) and lncRNAs (4%) (Figure 2C) in putative lncRNAs. FPKM was used to estimate the expression levels of mRNAs and lncRNAs (Figure 2D), and the results revealed that the overall expression level of mRNAs was higher than that of lncRNAs, and the expression level of most lncRNAs was low (FPKM ≤ 1). Moreover, the basic characteristics of mRNAs and lncRNAs, including transcript length, ORF length, and number of exons, were analyzed. The results showed that the total length and ORF length of mRNAs were longer than that of lncRNAs (Figures 2E,F), and the number of exons of mRNAs is more than that of lncRNAs (Figure 2G). In addition, significant differences were detected in the distribution of exons of mRNAs and lncRNAs. A 91.5% of lncRNAs had only one or two exons, while mRNAs had more exons and wider distribution. These results verified that the predicted mRNAs and lncRNAs were in line with general characteristics.

Sequence similarity and conservation are regarded as indicators of biological function. The conservative analysis of lncRNAs was assessed by using E-value < 1e-5. In longan, 28, 19, and 78 lncRNAs were sequentially conserved with *Arabidopsis thaliana* (15), *Malus domestica* (12), and *Citrus sinensis* (79), respectively (Supplementary Table 4), indicating that lncRNAs were less conserved among different species.

### Differential Expression and Functional Analysis of mRNA and lncRNA

**Figure 3:**
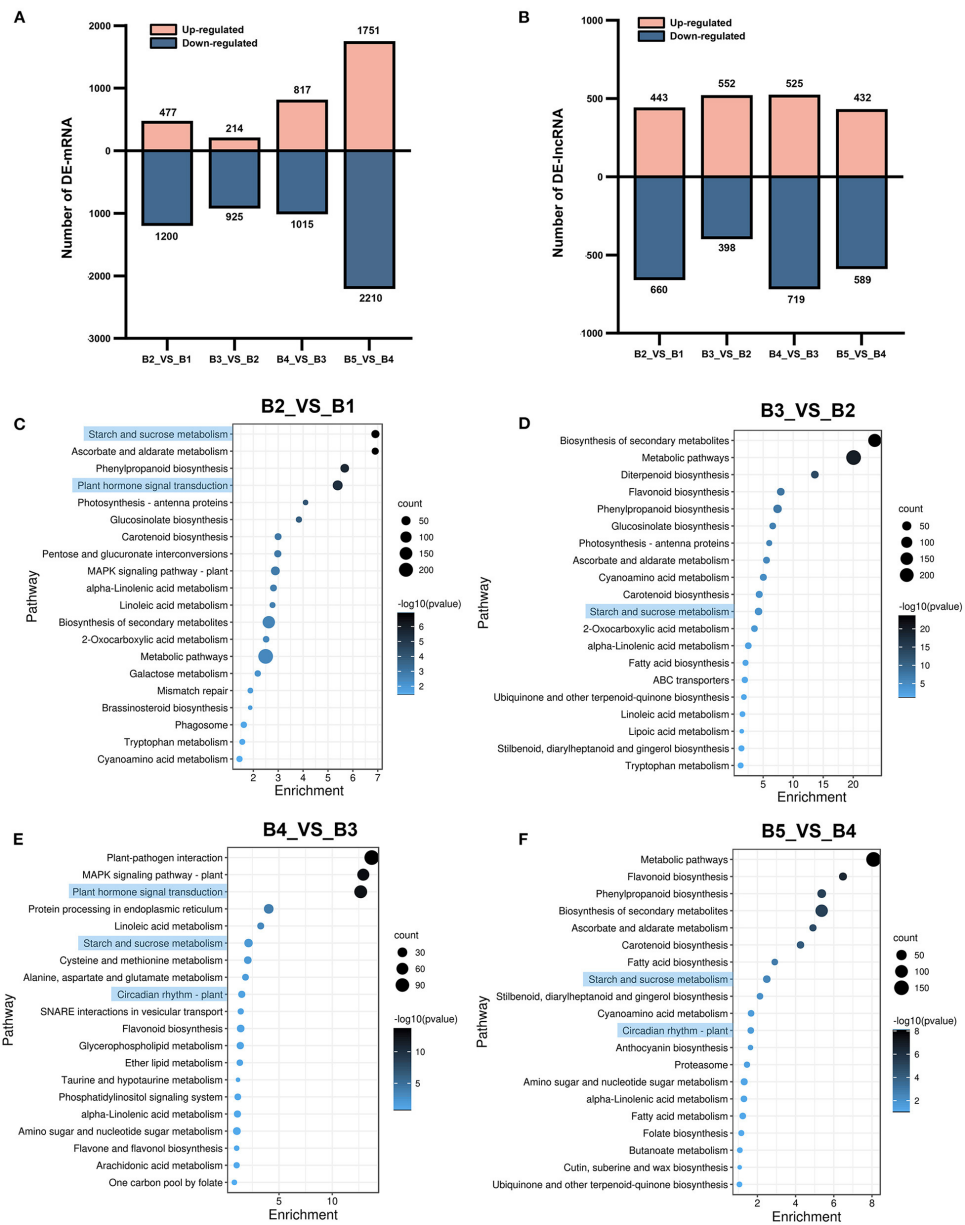
Up- and down-regulated numbers of DEmRNAs and DElncRNAs and KEGG enrichment analysis of DEmRNAs at different stages. **(A)** The number of up-regulated and down-regulated DEmRNAs. **(B)** The number of up-regulated and down-regulated DElncRNAs. **(C–F)** KEGG enrichment analysis of DEmRNAs in B2_*vs*_B1, B3_*vs*_B2, B4_*vs*_B3, and B5_*vs*_B4. The horizontal axis shows -log_10_(*p*). The vertical axis shows pathways of enrichment.

A total of 7,221 DEmRNAs were identified by time-series pairwise comparisons, which included 1,677 DEmRNAs in B2_*vs*_B1, 1,139 in B3_*vs*_B2, 1,832 in B4_*vs*_B3, and 3,961 in B5_*vs*_B4 (Figure 3A; Supplementary Table 5). A total of 3,238 DElncRNAs were identified, including 1,103 in B2_*vs*_B1, 950 in B3_*vs*_B2, 1,244 in B4_*vs*_B3, and 1,021 in B5_*vs*_B4 (Figures 3B,D; Supplementary Table 5). The transition from B4 stage to B5 stage resulted in more complex transcriptional changes in DEmRNAs than that in other stages.

A lncRNA/protein-coding gene regulatory pair consists of a controlled lncRNA and its regulated protein-coding gene. LncRNA has been reported to regulate the expression of protein-coding genes in both *cis*- and *trans*-regulatory mechanisms (Hu et al., 2016; Wei et al., 2019). On the basis of 3,238 DElncRNAs as studied, a total of 4,124 *cis*-regulated lncRNA-mRNA pairs were identified in the 100 kb range, including 1,947 DElncRNAs and 2,007 DEmRNAs (Supplementary Table 6). For lncRNA *trans*-targets, total of 13,840 regulatory pairs were identified, including 1,069 DElncRNAs and 1,482 DEmRNAs (Supplementary Table 6). These findings indicate that lncRNAs may demonstrate rich functions in *trans*-regulating their distant genes and co-location regulation.

Pathway-based analysis helps to further understand the biological function of genes. To explore transcriptional changes during floral induction, KEGG enrichment analysis was performed on DEmRNAs at different stages. Abundant metabolic and signaling pathways were enriched in all comparison groups, and pathways implicated in flowering, such as “starch and sucrose metabolism,” “circadian rhythms – plant,” and “plant hormone signal transduction”, were found. KEGG analysis showed that “starch and sucrose metabolism” was significantly enriched in the top 20 enriched pathways in all comparison groups. The “plant hormone signal transduction” pathway was significantly enriched in B2_*vs*_B1 and B4_*vs*_B3 (Figures 3C,E). In addition, the “circadian rhythm–plant” pathway was significantly enriched in B4_*vs*_B3 and B5_*vs*_B4 (Figures 3E,F). Three flowering-related KEGG pathways were also identified among the target genes of the DElncRNAs (Supplementary Table 6). The results showed that these three biological processes were transcriptionally active during floral induction in longan.

### Co-Expression Networks Reveal Modules Specific to Longan Floral Induction

**Figure 4:**
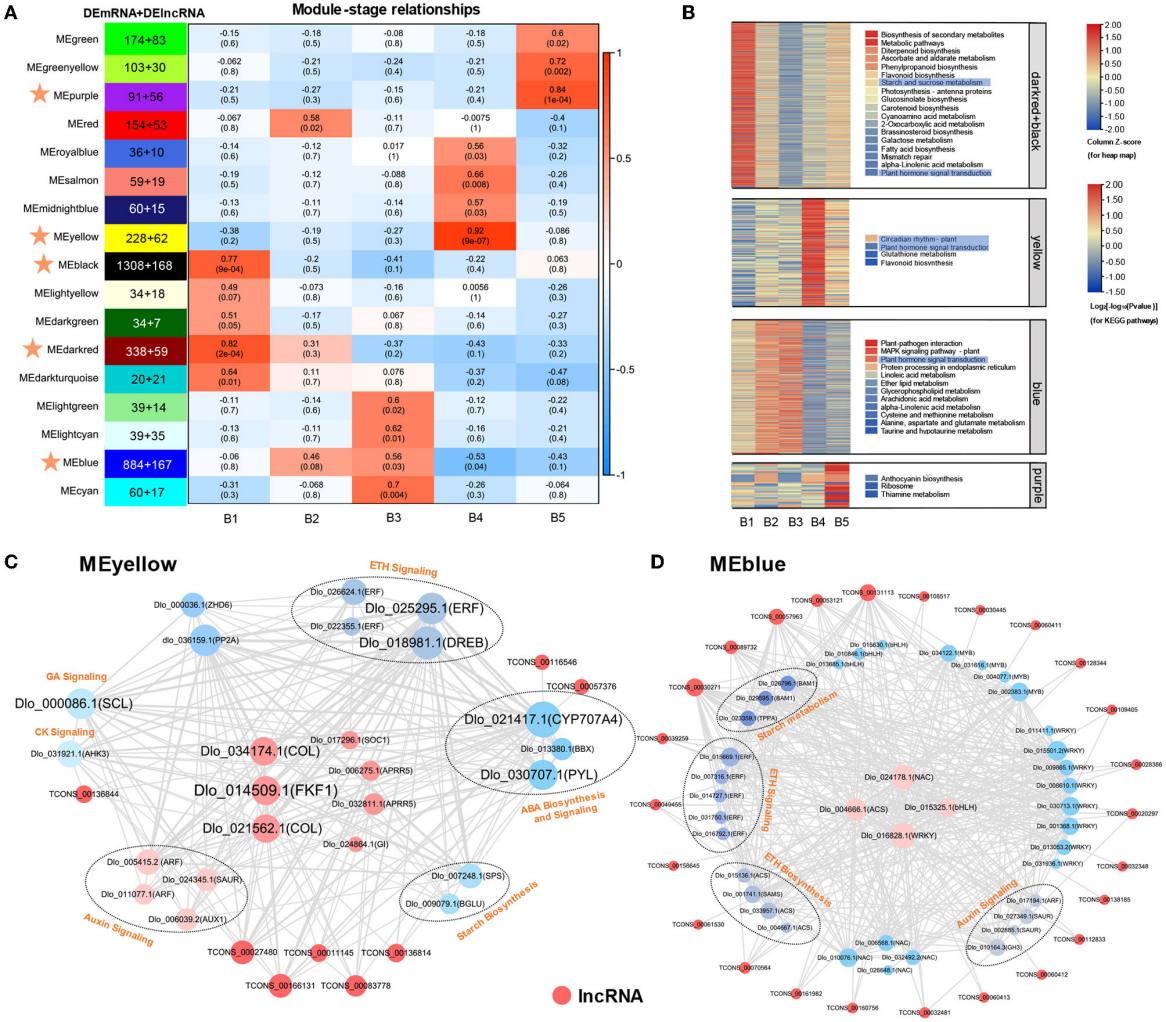
Co-expression network analysis of DEmRNAs and DElncRNAs. **(A)** Heatmaps showing correlation of module eigengenes at different stages of floral induction in longan. Pearson correlation coefficient is given in each module for the different stages and is colored from blue to red according to the scores. The MEpurple, MEyellow, MEblack, Medarkred, and MEblue modules (Pearson's correlation coefficient >0.75 or <-0.5 and *p* < 0.05) with stars are closely related to the floral induction in longan. **(B)** KEGG enrichment analysis and transcriptional changes of DEmRNAs in the MEpurple, MEyellow, MEblack, MEdarkred, and MEblue modules. The selected module KEGG enriched pathways are visualized by different colors. **(C,D)** Visualization of the central coexpression network in MEyellow and MEblue modules associated with B4 stage. The size of nodes indicates connectivity. The width of the line indicates the strength of the correlation.

To understand the dynamic changes of represented genes and systematically explore the potential regulatory function of lncRNA associated with longan floral bud differentiation, WGCNA was performed to analyze the co-expression relationship between 7,221 DEmRNAs and 3,228 DElncRNAs. Module–stage association analyses were performed to select stage-specific modules. A total of 17 different modules were obtained. MEblack, MEdarkred, MEyellow, MEblue, and MEpurple modules were highly correlated with different stages of longan floral induction (Pearson's correlation coefficient > 0.75 or < −0.5 and *p* < 0.05) (Figure 4A). KEGG enrichment (Figure 4B) and major expression center analyses were also conducted for the five modules. “Plant hormone signal transduction” was the most abundant pathway among the five modules. Closer inspection revealed that DEmRNAs in MEyellow and MEblue modules, which were highest associated with B4 stage, were significantly enriched in “circadian rhythm” and “plant hormone signal transduction,” suggesting that phytohormone and circadian rhythm may play a dominant role in B4 stage. The MEblack and MEdarkred modules were highest correlated with the B1 stage, and genes of “starch and sucrose metabolism” and “plant hormone signal transduction” pathways were significantly enriched in these two modules, indicating that sucrose and phytohormone played an important role in the regulatory network of B1 stage. KEGG enrichment analysis of DEmRNAs in these highly correlated 5 modules also showed the interaction between phytohormone signaling and circadian rhythms at the B4 stage, and the interaction between starch and sucrose metabolism and phytohormone signaling at the B1 stage (Figure 4B). The results of WGCNA were consistent with the results of KEGG enrichment analysis, suggesting that three regulatory pathways of starch and sucrose metabolism, phytohormone signaling and circadian rhythm may play important roles during longan floral induction.

Module membership (MM) represents the degree of connection based on the eigengene, which is a measure of a gene relative to the membership of the module and is used to screen the hub gene. DEmRNAs and DElncRNAs with MM > 0.75 were identified as hub genes. A total of 1,976 hub-DEmRNAs and 207 hub-DElncRNAs were identified (Supplementary Table 7). For example, highly connected hub genes in MEyellow module included “*two-component response regulator 5* (*APRR5*),” “*CONSTANS-LIKE* (*COL*),” “*FKF1*,” “*GI*,” and “*SOC1*,” which were well-known genes in circadian rhythm and photoperiod regulatory pathway during flowering. The central expression networks of MEyellow and MEred modules at the B4 stage were further analyzed (Figures 4C,D). The main hubs in the co-expression network of MEyellow module included rhythm-related genes (*COL, Dlo_034174.1, Dlo_021562.1*; *FKF1*), *ethylene-responsive transcription factor* (*ERF, Dlo_025295.1*), and ABA-degrading gene *CYP707A4* (Figure 4C), which were co-expressed with auxin, gibberellin, ethylene signaling, and sucrose biosynthesis genes, which were highly positively correlated. MEblue module contained more transcription factors, such as *bHLH, WRKY, MYB*, and *NAC*, which were highly negatively correlated, among them, *NAC* (*Dlo_024178.1*), *WRKY* (*Dlo_016828.1*), *bHLH* (*Dlo_015325.1*), and *ACS* (*Dlo_004666.1*) had the highest connectivity (Figure 4D). Similarly, MEblue hub genes were co-expressed with genes involved in phytohormone biosynthesis, signal transduction, and sucrose metabolism. Our findings revealed the crosstalk of three regulatory pathways during longan floral induction.

Interaction patterns between lncRNAs and mRNAs were also recognized within MEyellow and MEblue networks (Figures 4C,D). There were multiple interaction patterns between lncRNAs and mRNAs, such as one-to-one, one-to-many, and many-to-many. Hub-DElncRNAs are associated with multiple regulatory pathways. For example, TCONS_00166131 has the potential to regulate the photoperiod-related *COL* (*Dlo_034174.1, Dlo_021562.1*) and *FKF1* genes, the ethylene signaling-related *ERF* gene, and the sucrose biosynthesis-related *SPS* gene within the same module (Figure 4C), revealing that DElncRNAs may affect the expression of targeting genes in different pathways and serve as the regulator of gene co-expression networks. Transcripts, including DElncRNAs and DEmRNAs, in the same module have the same expression pattern and may also share related functions or biological pathways. Meanwhile, the results also showed that DElncRNAs had the potential for *trans*-regulating a spatial number of genes within modules, as opposed to tightly restricted *cis*-regulation.

### DEmRNAs in Sucrose Metabolism and Signaling Pathway

**Figure 5:**
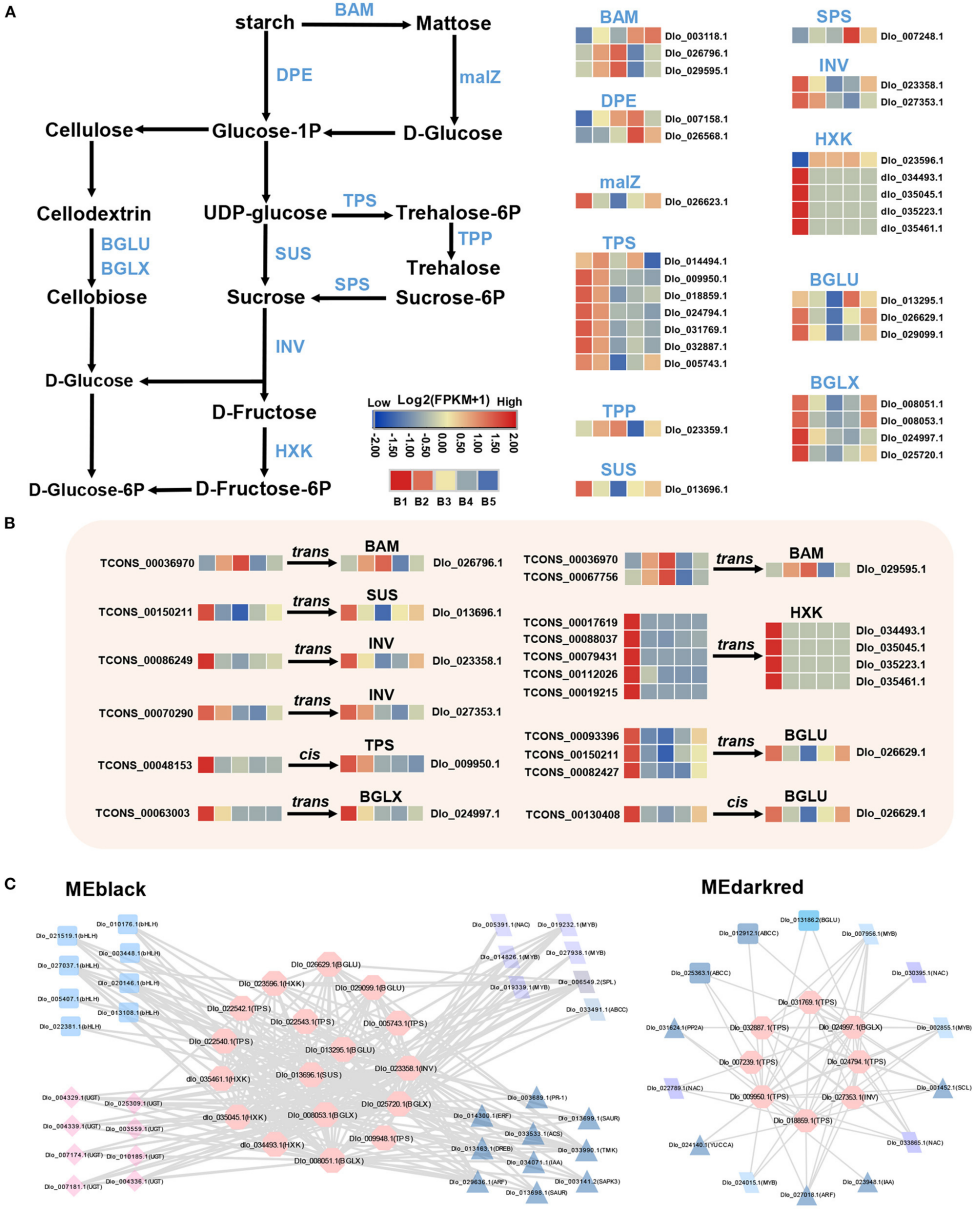
DEmRNAs and DElncRNAs in sucrose metabolism and signaling pathway. **(A)** Transcriptional changes in DEmRNAs associated with sucrose metabolism. **(B)** Expression profiles of hub-DElncRNAs and their target genes related to the sucrose metabolism based on the transcriptome data. Transcriptional change levels were normalized by log_2_transformed (FPKM + 1). **(C)** Co-expression network of genes involved in sucrose biosynthesis and metabolism in MEblack and Medarkred (pink nodes). Different nodes represent different types of genes, and edges connect co-expression genes.

A total of 30 DEmRNAs associated with carbohydrate biosynthesis and metabolism were identified. In sucrose biosynthesis, the expression levels of most DEmRNAs were up-regulated at the B1 stage, especially *sucrose synthase* (*SUS*) and *trehalose-phosphate synthase* (*TPS*). For starch degradation, a stronger up-regulation of DEmRNA expression was found at B3 and B4 stages, such as *beta-amylase* (*BAM*) and *4-*α*-glucanotransferase* (*DPE*) (Figure 5A). The transcript level of *sucrose phosphate synthase* (*SPS*) gene, which was one of the key enzymes for sucrose entry into metabolic pathways, was up-regulated at B4 stage. Most of the DEmRNAs involved in sucrose biosynthesis and metabolism were found in MEblack and MEdarkred modules, the majority of which included genes encoding *TPS*, β*-glucosidase* (*BGLU, BGLX*), *hexokinase* (*HXK*), and others (Figure 5C).

### DEmRNAs in Photoperiod and Circadian Clock Pathway

**Figure 6:**
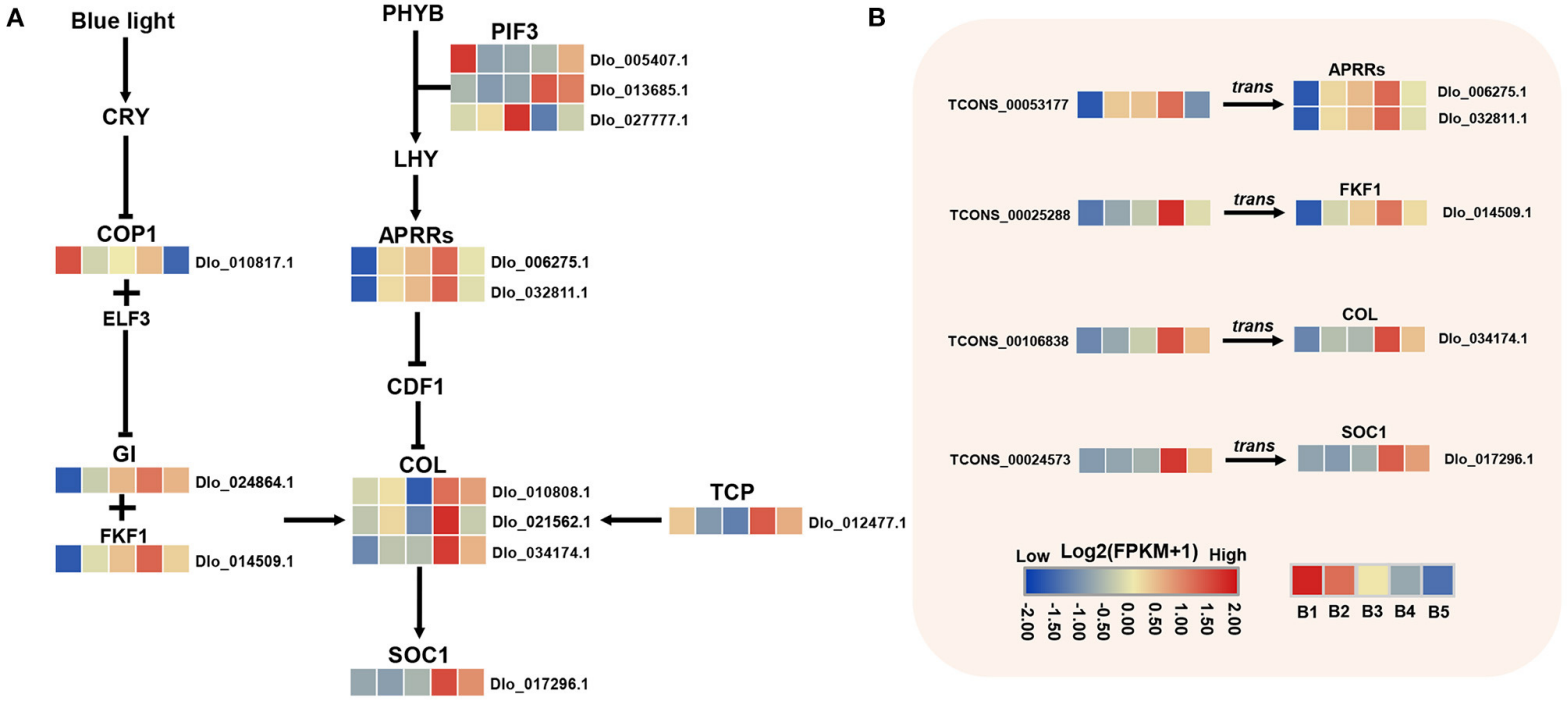
DEmRNAs and DElncRNAs in photoperiod and circadian clock pathways. **(A)** Transcriptional trends of DEmRNAs in photoperiod and circadian clock pathway and visualized as heatmaps. **(B)** DEmRNAs targeted by hub-DElncRNAs. Expression profiles of hub-DElncRNAs and their target genes related to photoperiod and circadian clock based on the transcriptome data. Transcriptional change levels were normalized by log_2_transformed (FPKM + 1).

The circadian pathway was one of the main enriched pathways by KEGG analysis. A total of 18 DEmRNAs were found in this pathway, most of them were unique to the MEyellow module and the expression levels were significantly up-regulated at the B4 stage (Figure 6A). Three *COL* genes were found located downstream of *FKF1* and *GI* genes, which had the same expression trend as *FKF1* and *GI*. The same up-regulated expression trend was also found for two orthologs of *Pseudo-response Regulator 5* (*PRR5, Dlo_006275.1, Dlo_032811.1*), as well as *SOC1*and *TCP*. A potential circadian regulatory pathway was constructed during floral induction in longan based on the identified DEmRNAs.

### DEmRNAs in Phytohormone Biosynthesis and Signal Transduction

**Figure 7:**
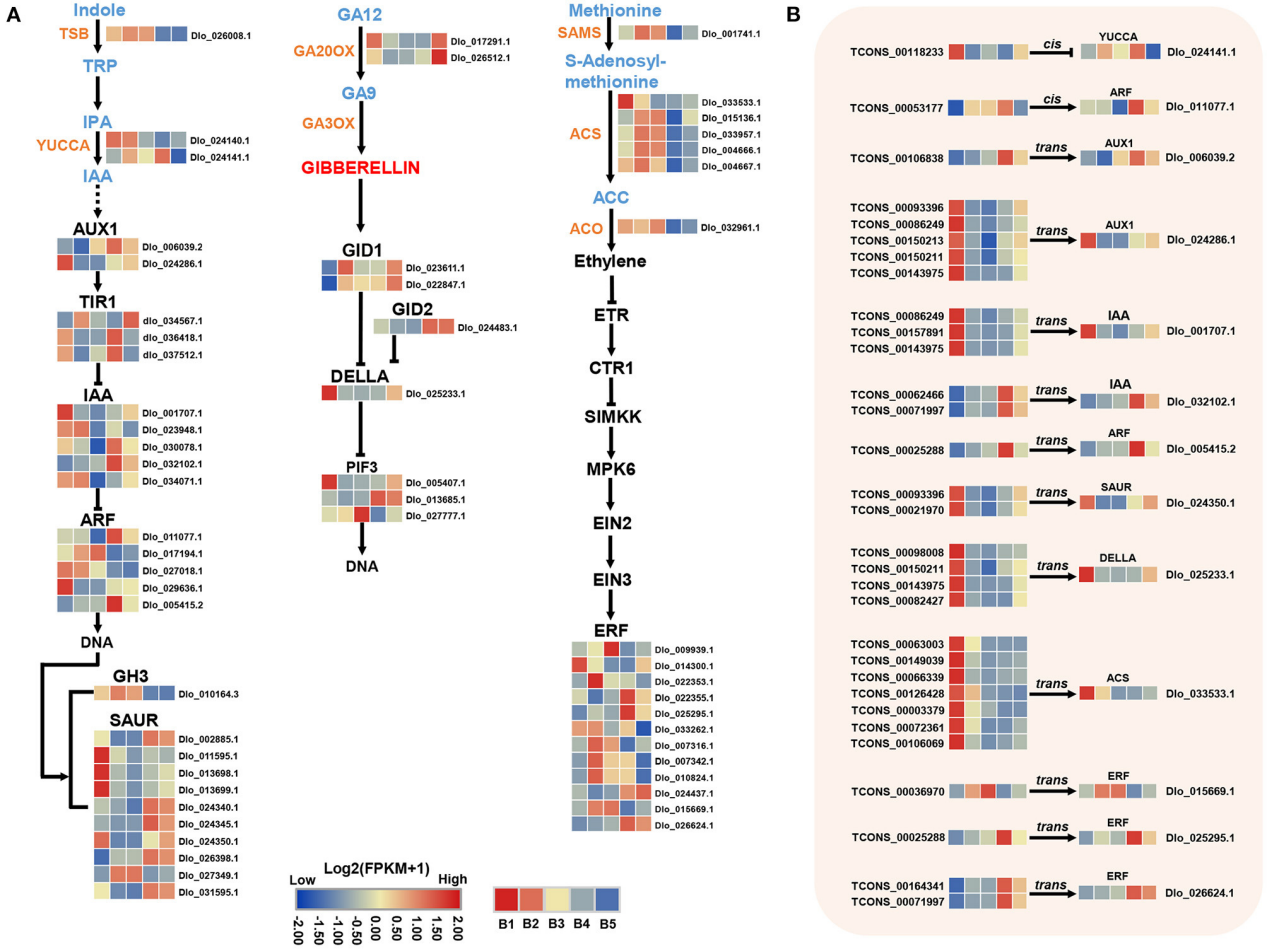
DEmRNAs and DElncRNAs in auxin, gibberellin (GAs), and ethylene (ETH) biosynthesis and signal transduction pathways. **(A)** Transcriptional changes of genes associated with auxin, GAs, and ETH at different stages visualized as heatmaps. **(B)** Expression profiles of hub-DElncRNAs and their target genes related to the phytohormone biosynthesis and signal transduction based on the transcriptome data. Transcriptional change levels were normalized by log_2_transformed (FPKM + 1).

Analysis of genes in hormone signaling pathways revealed at least two-fold significant changes in expression levels between different stages of floral induction. 29 and 10 DEmRNAs were identified in auxin and GA biosynthesis and signaling pathways, respectively, and 19 DEmRNAs were associated with ETH biosynthesis and signal transduction (Figure 7A). In the auxin pathway, two *YUCCA* genes, which positively regulate auxin biosynthesis, had opposite expression patterns. The expression of multiple *small auxin up RNAs* (*SAURs*) and *Aux/IAA* genes were down-regulated at the B3 stage. The expression of *ARF* (*Dlo_017194.1, Dlo_011077.1, Dlo_005415.2*) was up-regulated at B3 or B4 stage. In the GA biosynthesis pathway, the expression levels of two *GA20OX* genes were down-regulated at B3 and B4 stages, implying a decrease in the rate of gibberellin synthesis. In the ETH pathway, the rate-limiting enzymes of ethylene biosynthesis, namely, *1-aminocyclopropane-1-carboxylate oxidase* (*ACO*) and *1-aminocyclopropane-1-carboxylate synthase* (*ACS*), were both down-regulated at the B4 stage. The lower transcriptional levels of seven ethylene biosynthesis-related genes suggested that the ethylene accumulation may be reduced at B4 stage. Members of *ERF* gene family showed different expression patterns, among which three *ERF* genes, namely, *Dlo_009939.1, Dlo_007316.1, Dlo_015669.1*, had the same expression trend as biosynthetic genes.

### Potential Hub-DElncRNAs With Their Target Genes in Three key Pathways

To determine the role of 229 Hub-DElncRNAs during longan floral induction, their co-localized and co-expressed protein-coding genes were predicted; 33 hub-DElncRNAs were related to sucrose metabolism, circadian rhythm, and phytohormone biosynthesis and signaling pathways, and the specific lncRNA-mRNA regulatory relationship was shown in Figures 5B, 6B, 7B; Supplementary Table 8.

**Figure 8:**
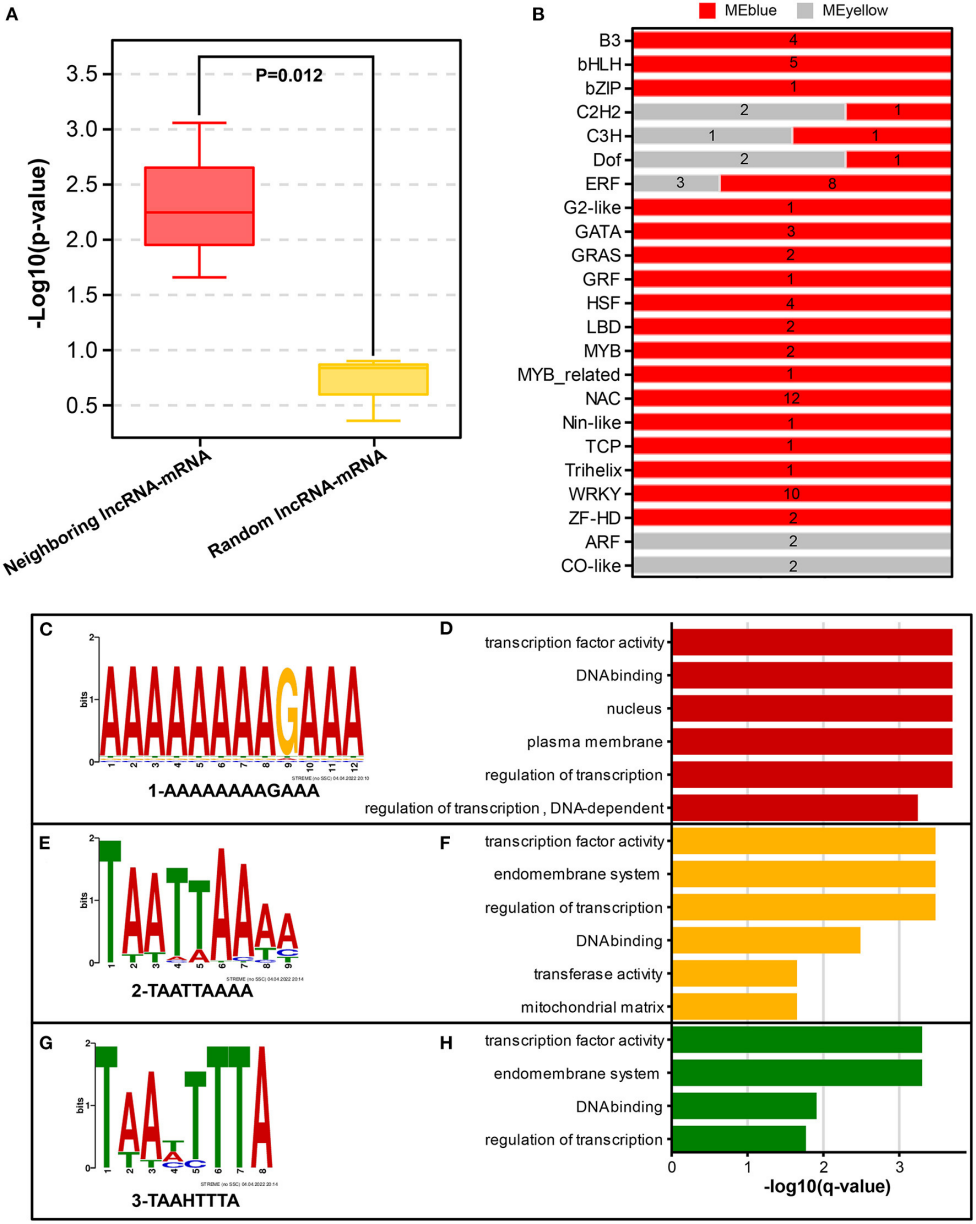
Potential regulatory modes of lncRNAs. **(A)** Comparison of co-expression correlations between neighboring lncRNA-mRNA and random lncRNA-mRNA groups. Significant differences between the two groups were analyzed using Duncan's test. **(B)** Quantity distribution of transcription factors (TFs) included in two example modules (MEblue and MEyellow). **(C,E,G)** Three representative binding motifs in lncRNA sequences from MEyellow and MEblue modules. **(D,F,H)** Functional analysis of 3 representative lncRNA-binding motifs using GOMO tool.

Correlation analysis of neighboring lncRNA–mRNA pairs (Supplementary Table 9) revealed that three Hub-DElncRNAs (TCONS_00048153, TCONS_00130408, TCONS_00053177) and their neighboring DEmRNAs (*TPS, Dlo_009950.1*; *BGLU, Dlo_026629.1*; *ARF, Dlo_011077.1*) were positively correlated (Pearson's correlation coefficient ≥ 0.58, *p* ≤ 0.05), and one neighboring lncRNA-mRNA pair had a negative correlation, suggesting that hub-DElncRNAs regulated neighboring protein-coding genes *via cis*-regulatory manner, mainly in a positive manner. In addition, neighboring lncRNA–mRNA pairs may be activated by the same promoter or enhancer for simultaneous transcriptional expression (Ye et al., 2022). To investigate the specific regulatory mechanism of the positive correlation, random lncRNA–mRNA pairs were used as the comparison group for analyzing. The results showed that the correlations of the three neighboring lncRNA–mRNA pairs were statistically significant compared with random lncRNA–mRNA pairs (*p*=0.012) (Figure 8A), indicating that neighboring lncRNA–mRNAs shared the same promoters or enhancers partly.

To further elucidate the *trans*-regulatory roles of lncRNAs in the three pathways, a total of 31 hub-DElncRNAs with *trans*-regulatory potential were identified, among them, there were 16 sucrose metabolism-related, 5 circadian rhythm-related, and 29 hormone signal transduction-related lncRNA-mRNA pairs. Correlation analysis showed that these lncRNA–mRNA pairs had positive correlations, suggesting that hub-DElncRNAs have positive effects on these distant genes.

In the detection of the regulatory relationship between lncRNA and mRNA, 15 hub-DElncRNAs were found to target multiple genes, and 16 hub-DElncRNAs had only one target gene. Sucrose metabolism-related genes (*BAM, Dlo_026796.1*; *SUS, Dlo_013696.1*, and others), four photoperiod-related genes (*FKF1, APRR5, COL*, and *SOC1*), and four hormone signal transduction-related genes (*AUX1, Dlo_006039.2*; *ARF, Dlo_005415.2*; *ERF, Dlo_025295.1, Dlo_015669.1*) were target genes and they were regulated by a single hub-DElncRNA, respectively. One-to-many and many-to-many regulatory relationships between hub-DElncRNAs and potential target genes were also observed. For example, TCONS_00025288 had three targets, the rhythm gene *FKF1, ARF* (*Dlo_005415.2*), and *ERF* (*Dlo_025295.1*). These results suggested that lncRNAs may act as the regulator in different pathways and play an important role in the regulatory network of floral induction.

LncRNAs have also been reported to regulate gene expression by binding to transcription factors (TFs) directly (Long et al., 2017; Ye et al., 2022). Using the MEyellow and MEblue modules as examples for predicting intra-module transcription factors, several highly connected TFs (including *bHLH, WRKY, ERF*, and *NAC*) were found were co-expressed with sucrose metabolism, photoperiod, and hormone-related genes in the co-expression network (Figure 8B). LncRNA has the possibility to regulate 3 putative pathway-related genes by binding to these TFs. Therefore, the identification of binding motifs in lncRNAs will help predict the interactions between lncRNAs and their target TFs. “AAAAAAAAGAAA,” “TAATTAAAA,” and “TAAHTTTA” were identified as three major binding motifs of lncRNA sequences (Figures 8C,E,G; Supplementary Table 10). GOMO analysis (Figures 8D,F,H; Supplementary Table 10) showed that the three motifs had transcription factor activity, DNA binding, and transcriptional regulatory functions, indicating that lncRNAs had great potential to interact with TFs through both transcriptional and post-transcriptional regulatory ways. The TF families co-expressed with lncRNAs in modules are worthy of further study.

### qRT-PCR Validation of DEmRNAs and DElncRNAs

**Figure 9:**
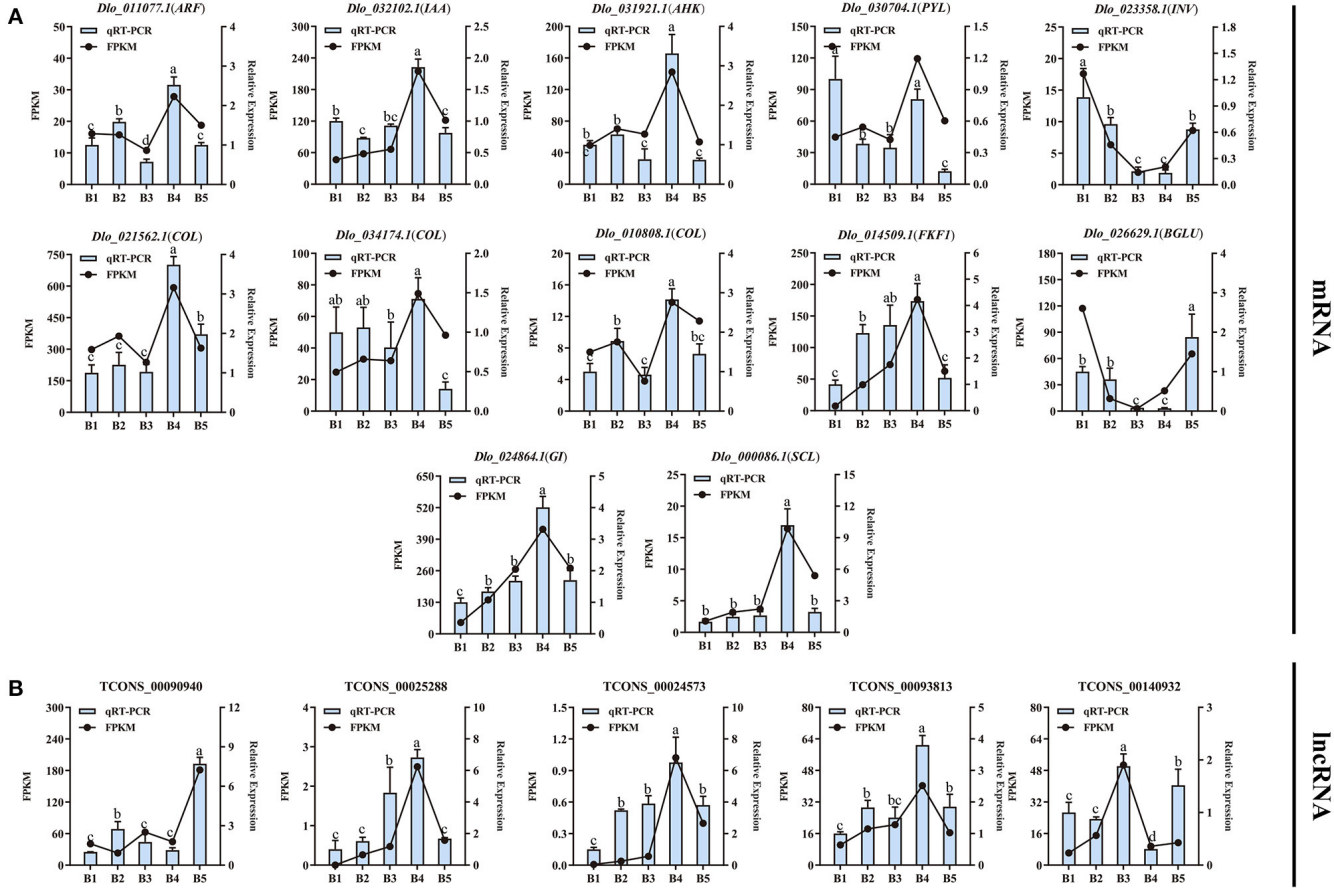
Consistency analysis of RNA-Seq and qRT-PCR data. **(A)** Verification of the consistency of RNA-Seq data and qRT-PCR of 12 DEmRNAs. **(B)** Verification of the consistency of RNA-Seq data and qRT-PCR of 5 DElncRNAs. The different letters (a, b, c, d) show significant differences at the *p* < 0.05 level based on Duncan's test.

The transcript abundances of 12 DEmRNAs and 5 DElncRNAs involved in three presumptive pathways were analyzed by qRT-PCR (Figures 9A,B; Supplementary Table 11). The qRT-PCR results showed that expression levels of *ARF, IAA, AHK, COL, FKF1, GI*, and *SCL* were significantly up-regulated at the B4 stage and were consistent with the transcriptome data. The same variation trends for the DEmRNAs were well shown between qRT-PCR and FPKM values, indicating the reliability of the RNA-Seq data. The expression levels of 5 DElncRNAs were also analyzed by qRT-PCR. The expression levels of DElncRNAs were consistent with their target genes. For example, the expression level of TCONS_00025288 was consistent with its target gene *FKF1*, showing a positive correlation.

## Discussion

### Three Important Regulatory Pathways During Floral bud Induction of Longan

In this study, transcriptome sequencing was performed to study the transcriptional changes and regulatory pathways during floral induction in longan. From the results of KEGG enrichment and WGCNA analysis of DEmRNAs, three pathways (“starch and sucrose metabolism,” “circadian rhythm,” and “plant hormone signal transduction”) were found to play an important role during floral bud induction. At B1 and B2 stages, vigorous sucrose metabolism was observed, and phytohormone signal transduction was also found to play an important role in this period. In Fujian Province, B1 and B2 stages (September and October) are the time when autumn shoots grow and get maturity (Yuan, 2016). The level of endogenous nutrient accumulation is directly related to the development of autumn shoots in longan (Li et al., 2013). Therefore, our results confirm that endogenous nutrition and hormones are important for the growth of fruiting branches. In addition, it is the fruiting season for “LDB” longan in September and October. Developing fruits provide a strong sink for photoassimilates. It is thought that depletion of photoassimilates, especially carbohydrates from the buds, may affect the number of floral buds (Goldschmidt et al., 1985; Goldschmidt, 1999; Shalom et al., 2014). Therefore, it is speculated that the sucrose accumulated at B1 stage may be partly supplied to the developing fruits. At B3–B4 stage, DEmRNAs were significantly enriched in all three pathways, especially genes in the circadian rhythm pathway were strongly up-regulated. November and December (B3 and B4 stages) were thought to be the period of physiology differentiation of floral bud induction (Yuan, 2016), when apical buds sense environmental changes, such as low temperature and short photoperiod, to ensure the stability of floral induction (Suttitanawat et al., 2012). Our results were consistent with the characteristics of physiological differentiation of floral induction, suggesting that apical buds respond to external environmental signals and initiate the internal physiological changes in this period. Meanwhile, our results also showed that sucrose and hormones were necessary for the physiological differentiation of floral buds. Previous studies have highlighted that sucrose-mediated signaling is interconnected with hormone-mediated floral induction, possibly as an additional part of flowering initiation (Wang et al., 2021).

### Starch and Sucrose Metabolism-Related Genes Mediate Floral Induction in Longan

Carbohydrates are essential for plant growth and development, including flowering (Gibson, 2005; Matsoukas, 2014). B1 stage-related MEdarkred and MEblack modules significantly enriched a large number of sucrose metabolism-related genes (such as *SUS, INV*, and *HXK*), indicating that apical buds of longan mainly provided nutrients in the form of sucrose during B1 and B2 stages. Notably, four homologous genes of *HXK*, which act as sugar sensors sensing sugar levels and phosphorylation status and transmitting signals (Jang and Sheen, 1994; Rolland and Sheen, 2005), were only expressed at the B1 stage with spatiotemporal specificity, suggesting that *HXK* genes in longan participated in the sucrose-induced signal transduction at B1 stage and responded specifically to the growth and development of autumn shoots. Trehalose-6-phosphate (T6P) has been proposed as a proxy for carbohydrate status in plants (Wahl et al., 2013). T6P level is positively correlated with sucrose level, and an increase in T6P level leads to an increase in sugar metabolism and signaling which initiates floral induction (Lastdrager et al., 2014). In our study, the expression levels of *TPS* genes, which were key genes involved in T6P biosynthesis pathway, were up-regulated during B1–B2 stage, suggesting that both sucrose and T6P act as the proxy for the carbohydrate status in fruiting branches of longan.

Starch is an important energy storage form that can be hydrolyzed into sugar for the utilization of floral buds (Li et al., 2013). The expression levels of key enzyme genes related to starch degradation and sucrose biosynthesis, such as *BAM, DPE*, and *SPS*, were up-regulated at B3 or B4 stage, leading to starch degradation and sucrose accumulation, which provided energy for the physiological differentiation of floral induction. A similar result was reported in apple (Xing et al., 2015). *BAM* genes (*AtBAM1, AtBAM3*) are required for meristem function in *Arabidopsis* (DeYoung et al., 2006). In the present study, *BAM* genes (*Dlo_026796.1, Dlo_029595.1, Dlo_003118.1*) had higher expression at B3 and B4 stages, suggesting that they may be involved in maintaining the normal development of longan floral bud meristem. Sugar levels and metabolism-related gene expression profiles revealed that sucrose is the initiation signal in apple floral induction, and sucrose accumulation has a positive influence on floral induction and development (Xing et al., 2015; Du et al., 2017). This suggests that sucrose plays an essential role in the regulation of bud growth and carbohydrate levels that induce sugar signal and promote floral induction in longan.

### Circadian Clock and Photoperiod-Related Genes Play key Roles During Floral Induction in Longan

Plants possess a circadian clock that enables them to coordinate internal biological events with changes in external rhythm (Imaizumi, 2010). The photoperiod pathway mediates light and temporal information from the environment into the regulation of flowering time (Cheng and Wang, 2010). In perennial woody plants, the circadian clock may sense seasonal cues and induce flowering seasonally (Jia et al., 2014). In this study, *FKF1, GI, APRR5* (*Dlo_006275.1, Dlo_032811.1*), *COL*, and *SOC1* genes exhibited higher transcript accumulation levels at the B4 stage, indicating that circadian clock and photoperiod-mediated flowering induction is an important pathway in the flowering regulatory network in longan. *FKF1* and *GI* play important roles in circadian oscillation and flowering time regulation (Tootle et al., 2003; Mishra and Panigrahi, 2015). A previous report suggested that longan *GI* and *FKF1* were flowering promoters and may regulate the floral bud physiological differentiation by affecting endogenous auxin synthesis and transport (Huang et al., 2017). Consistent with that, in this study, *FKF1* was found to act as a central hub gene co-expressed with auxin signaling-related genes in the B4-related MEyellow module. In addition, two *CONSTANS-LIKE* (*COL*) family members (*Dlo_021562.1, Dlo_034174.1*) were identified in the photoperiod pathway, whose homolog (*AtCOL4, AtCOL9*) had an important role in regulating flowering time in *Arabidopsis* (Cheng and Wang, 2010; Steinbach, 2019). It has been reported that the deletion of a pair of *COL* genes may affect flowering time in lychee (Hu et al., 2022). *COL* is the central regulator in the photoperiod pathway, down-stream of the circadian control (Jang et al., 2008). In this study, longan two *COL* genes were found to be co-expressed with *FKF1* and *GI*, suggesting they may be under the direct regulation of *FKF1* and *GI* the same as in *Arabidopsis*. Moreover, *CO* was reported to be directly up-regulating the floral-pathways integrator *FT* gene (Valverde et al., 2004). However, the transcript of *FT* was not detected in our transcriptome database. Instead, *SOC1* was detected. *SOC1* is also a floral-pathways integrator, which is directly activated by *CO* in long photoperiod in *Arabidopsis* (Lee and Lee, 2010) and litchi (Zhang et al., 2014). These results suggest that *SOC1* may be the key flowering integrator in longan and *COL* (*Dlo_021562.1, Dlo_034174.1*) may control photoperiod floral induction by activating the expression of *SOC1*. *APRR5* coordinates and positively regulates flowering time by regulating the *CO*-dependent photoperiod pathway (Nakamichi et al., 2005, 2007). Two *APRR5* genes showed strong up-regulation at B4 stage, suggesting that they had a potential function in longan floral induction. Overall, key genes related to circadian clock and photoperiod pathway were identified in longan, circadian clock, and photoperiod regulatory pathway, which may play a key role in seasonal floral induction in longan.

### Hormone Biosynthesis and Signaling Mediate Floral Induction in Longan

Phytohormones are signaling molecules induced by plant cells to receive specific environmental signals and regulate multiple aspects of plant physiological responses and development at low concentrations (Davis, 2009). Auxin is a key factor in inducing flower formation (Cho et al., 2017). The Medarkred, Meblack, MEyellow, and MEblue co-expression center networks all contained multiple auxin signaling-related genes, indicating that auxin plays an important role in different stages of longan floral induction. At the B4 stage, three auxin signaling-related genes (*ARF, Dlo_011077.1, Dlo_005415.2*; *AUX1, Dlo_006039.2*) in the co-expression center network of MEyellow modules showed increased expression. Moreover, three auxin signaling-related genes (*SAUR, Dlo_027349.1, Dlo_002885.1*; *GH3, Dlo_010164.3*) in the MEblue module showed decreased expression. Both *GH3* and *SAUR* genes have a negative feedback regulation of auxin signaling (Staswick, 2005; Nektarios et al., 2013). Several studies have indicated that high concentrations of IAA may have the effect of promoting the physiological differentiation of floral buds in longan (Su et al., 1997; Yang et al., 2019). Our results confirm that auxin may promote the floral bud differentiation in longan. The identified candidate gene was *ARF* (*Dlo_005415.2*), whose homolog (*AtARF2*) showed late flowering in *Arabidopsis* mutants (Okushima et al., 2005). As the core gene of auxin signal transduction pathway, *ARF* plays a synergistic role in multiple pathways mediated by auxin transduction signal (Peng et al., 2020). In addition, *ARFs* represent a point of cross-talk between ethylene and auxin signaling (Li et al., 2006). *ARF* (*Dlo_005415.2, Dlo_011077.1*) as hub genes were co-expressed with photoperiod-related genes (*COL, FKF1*), sucrose biosynthesis gene (*SPS*), and *ERF* genes (Figure 4C), indicating that *ARF* genes have potential regulatory capacity of crosstalk in synergistic key pathways related to longan floral induction.

In the model plant *Arabidopsis*, GA plays an active role in flowering through a GA-dependent signaling pathway (Mutasa-Gottgens and Hedden, 2009). Some perennial woody fruit trees, including mango (Nakagawa et al., 2012) and apple (Wilkie et al., 2008; Zhang et al., 2019), have been shown to have a negative response for GAs in floral bud formation. Also, several studies have documented that the floral induction in longan requires low levels of GAs (Huang, 1996; Wang et al., 2015). As a key negative regulator of GA signaling, *DELLA* gene was down-regulated during B3 and B4 stages, which was inconsistent with previous results of the inhibition function of GAs in longan. Therefore, it may be a different regulatory mechanism of GAs with *Arobidopsis* during flowering initiation in longan, which needs more investigation.

Ethylene is widely distributed in plants and closely related to the process of plant flower formation (Johnson and Ecker, 1998). However, unlike auxin and GA, little attention has been paid to ETH function in floral induction of woody fruit trees. In contrast to other stages, DEmRNAs in ETH biosynthesis maintained low expression levels at the B4 stage. This finding showed that reduced ETH level at the B4 stage may favor the process of floral bud induction. *ERF* is the key transcription factor in ETH signaling pathway (Zhang et al., 2020a). In the central co-expression network of the MEblue module, *ERF* (*Dlo_015669.1, Dlo_007316.1*) genes were co-expressed with ETH biosynthesis-related genes, all of which showed down-regulated expression levels at B4 stage. Therefore, these two *ERFs* may function by responding to decreased ethylene levels and sense external environmental changes at B4 stage in longan.

### Crosstalk Among Three Pathways

Longan floral induction requires coordination between external environmental conditions and various internal physiological factors, such as hormones and carbohydrates (Lin and Lu, 1992). It has been reported that exogenous auxin resistance was shown in *Arabidopsis hxk*/*gin2* mutants, while auxin-resistant mutants were insensitive to high glucose levels (Rolland and Sheen, 2005). In addition, the expression levels of auxin and trehalose biosynthesis-related genes in buds were simultaneously affected by fruit load in citrus (Shalom et al., 2014). These studies suggest a link between sugar and hormones. In our study, sucrose metabolism-related genes were co-expressed with hormone signaling-related genes in the MEdarkred and MEblack networks, revealing the crosstalk between these two pathways. An interaction of sucrose and hormones could take place at B1 and B2 stages, affecting the growth and development of autumn shoots of longan.

WGCNA co-expression network analysis revealed that three putative key pathways interacted at the B4 stage to form a regulatory network. Sugars are inducers of signals. Exogenous sucrose application can induce the expression of circadian clock-related genes, such as *AtGI* and *AtTOC1* (Dalchau et al., 2011). Endogenous sugars (such as sucrose and glucose) produced by photosynthesis can entrain circadian clock-related genes in *Arabidopsis* (Román et al., 2021). These findings suggest that sugars have crosstalk with circadian-related genes during flowering in plants. More evidence supporting the close association of flowering with sugar-related genes has been reported (Seo et al., 2011). The late-flowering phenotype of the *CO*-defective mutant is partly restored by sucrose feeding in darkness, suggesting that sucrose-mediated signals are incorporated into the photoperiod flowering pathway (Roldán et al., 1999). Consistent with that, in our study, rhythm genes in MEyellow and MEblue modules as central hubs were co-expressed with sucrose metabolism-related genes. Sucrose accumulation at the B4 stage may induce the upregulation of circadian rhythm-related genes. In addition, previous studies showed that the expression of a subset of genes involved in auxin biosynthesis, perception, and signaling was controlled by the clock (Covington and Harmer, 2007). *DlGI* and *DlFKF1* transgenic *Arabidopsis* showed that *DlGI* and *DlFKF1* may regulate auxin biosynthesis and transport (Huang et al., 2017). Consistent with this, *FKF1, GI*, and auxin-related genes (*ARF, Dlo_011077.1, Dlo_005415.2*) were co-expressed and showed high correlation at B4 stage, suggesting that circadian rhythm genes interact with the hormones signal transduction pathway and participate in longan seasonal floral induction.

### LncRNAs Regulate Longan Floral Induction by Coordinating the Expression of Genes in Three key Pathways

Comparing the co-expression of neighboring and random lncRNA–mRNA pairs revealed that lncRNAs primarily regulate neighboring gene expression in a positive *cis*-regulatory manner. This *cis*-regulatory effect has also been observed in other plants, such as *Oryza sativa, Vitis vinifera*, and *Populus trichocarpa* (Zhang et al., 2020b; Huang et al., 2021; Ye et al., 2022). In contrast to the boundedness of neighboring *cis*-regulatory functions, the *trans*-regulatory functions of lncRNAs at the genome-scale theoretically have an infinite set of target genes (Ye et al., 2022). Therefore, studying lncRNA–mRNA pairs expressed in a similar pattern will provide more information. In this study, based on the fact that the number of co-transcribed lncRNA–mRNA pairs was much more than that of *cis*-regulated lncRNA–mRNA pairs, it is indicated that *trans*-regulation of lncRNA is the main way to regulate three key pathways.

Some lncRNAs have been reported to target significantly differentially expressed mRNAs with different functions as well as specifically target functional mRNAs, and lncRNAs may act as regulators (Xue et al., 2019). The same results were shown in our study. For instance, two lncRNAs (TCONS_00036970, TCONS_00063003) positively regulate the expression of both ethylene and sucrose metabolism-related genes (*BAM, Dlo_026796.1, Dlo_029595.1*; *ERF, Dlo_015669.1*; *ACS, Dlo_033533.1*; *BGLX, Dlo_024997*). DEmRNAs analysis above has shown the interaction among sucrose metabolism, rhythms, and hormones. These results provide more evidence that lncRNAs participate in the regulatory network of floral induction by positively regulating the expression of key players of three pathways. In addition, lncRNAs may bind to flowering TFs through binding motifs and thus affect floral bud differentiation. Similar results also appeared in the study of *Populus trichocarpa* (Ye et al., 2022). This specific mechanism of lncRNA–mRNA action needs to be further studied.

### Hypothetical Model of Regulatory Pathways, key Genes and LncRNAs During Floral bud Induction in Longan

**Figure 10:**
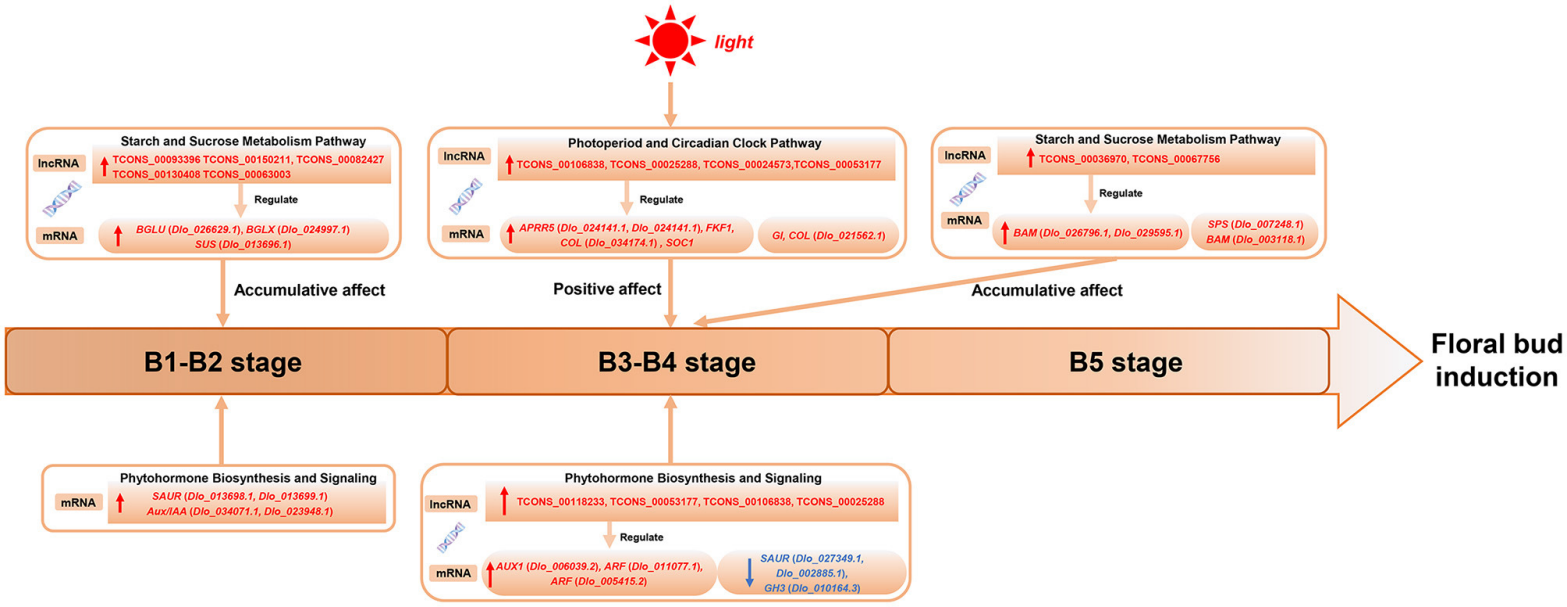
A hypothetical model showing three genetic pathways regulating floral bud induction in longan.

A hypothetical model for the regulation of longan floral induction *via* sugar, circadian, and hormone signaling and their crosstalk are shown in Figure 10; 21 hub-DEmRNAs and 12 hub-DElncRNAs involved in the three pathways are listed. In conclusion, at B1 stage the expression of sucrose metabolism-related genes (*BGLU, Dlo_026629.1*; *BGLX, Dlo_024997.1*; *SUS*) and auxin signaling negative feedback factors (*SAUR, Dlo_013698.1, Dlo_013699.1*; *Aux/IAA, Dlo_034071.1, Dlo_023948.1*) were up-regulated, which may be related to the growth and maturation of autumn shoots and fruit load. During physiological differentiation of floral buds, especially at the B4 stage, the circadian rhythm-related genes (*APRR5, FKF1, GI, COL, SOC1*) were co-expressed with sucrose metabolism-related genes (*SPS, BAM*) and auxin signal transduction-related genes (*ARF, Dlo_011077.1, Dlo_005415.2*; *AUX1, Dlo_006039.2*), actively participating in the regulation of the floral bud induction. High expression of auxin signaling genes may be associated with sucrose accumulation and positive response to circadian rhythm signals. Meanwhile, 12 hub-DElncRNAs in the three pathways were also showed, which affected gene expression in a positive regulatory manner and strengthened the connection of the three pathways.

## Data Availability Statement

The original contributions presented in the study are publicly available. This data can be found here: https://www.ncbi.nlm.nih.gov/, PRJNA830603.

## Author Contributions

LZ and SZ conceived the study and designed the experiments. FL, YZ, XW, SY, and TF conducted experiments and analyzed data. FL wrote the manuscript. All authors read and approved the final manuscript.

## Funding

This work was financially supported by the Fujian Agriculture and Forestry University Science and Technology Innovation Fund (KFA20028A) and the Fujian Agriculture and Forestry University Outstanding Graduate Student Fund (1122YS010 and 1122YS01008).

## Conflict of Interest

The authors declare that the research was conducted in the absence of any commercial or financial relationships that could be construed as a potential conflict of interest.

## Publisher's Note

All claims expressed in this article are solely those of the authors and do not necessarily represent those of their affiliated organizations, or those of the publisher, the editors and the reviewers. Any product that may be evaluated in this article, or claim that may be made by its manufacturer, is not guaranteed or endorsed by the publisher.

## Footnotes

^1^ https://cpc.cbi.pku.edu.cn/

^2^ http://pfam.xfam.org/

^3^ https://www.bioinfo.org/software/cnci/

^4^ https://www.genenames.org/

^5^ http://greenc.sequentiabiotech.com/wiki/Main_Page

^6^ https://meme-suite.org/meme/tools/streme

^7^ https://meme-suite.org/meme/tools/gomo

^8^ http://planttfdb.gao-lab.org/prediction.php
